# Efficacy and safety of bexarotene combined with photo(chemo)therapy for cutaneous T‐cell lymphoma

**DOI:** 10.1111/1346-8138.15310

**Published:** 2020-03-18

**Authors:** Akimichi Morita, Chiharu Tateishi, Shinnosuke Muramatsu, Ryouji Kubo, Eri Yonezawa, Hiroshi Kato, Emi Nishida, Daisuke Tsuruta

**Affiliations:** ^1^ Department of Geriatric and Environmental Dermatology Nagoya City University Graduate School of Medical Sciences Nagoya Japan; ^2^ Department of Dermatology Osaka City University Graduate School of Medicine Osaka Japan

**Keywords:** bexarotene, cutaneous T cell lymphoma, Japanese, phototherapy, treatment outcome

## Abstract

Cutaneous T‐cell lymphoma (CTCL) is a chronic condition with low malignancy. International treatment guidelines for CTCL are widely followed in Europe and the USA. Combination therapy with therapeutic agents for CTCL and phototherapy is effective on the basis of European data. The efficacy and safety of combination therapy for Japanese CTCL patients are not established. We investigated the efficacy and safety of combination therapy with photo(chemo)therapy and bexarotene in Japanese CTCL patients. Twenty‐five patients received daily oral bexarotene (300 mg/m^2^ body surface), followed by bath‐psoralen plus ultraviolet (UV)‐A (PUVA) or narrowband UV‐B. Treatment results were evaluated using the modified Severity‐Weighted Assessment Tool (mSWAT) and the Physician Global Assessment of Clinical Condition (PGA) up to week 24. Safety was also assessed. Twenty‐four weeks after initiating treatment, the total response rate was 80.0% (mSWAT) and 84.0% (PGA). Response rates did not differ when stratified by disease stage. Number of days (mean ± standard deviation) for time to response, duration of response and time to progression determined by the mSWAT were 20.7 ± 9.62, 117.0 ± 43.0 and 163.6 ± 28.8, respectively. T‐helper 2 chemokine levels in patients at stage IIA or more decreased significantly at weeks 12 and 24. All patients experienced adverse events and adverse drug reactions. Serious adverse drug reactions included sepsis, anemia and congestive cardiac insufficiency (*n* = 1 each). Other adverse drug reactions were of mild to moderate severity. Combination therapy with bexarotene and PUVA was safe and effective in Japanese CTCL patients.

## Introduction

Primary cutaneous T‐cell lymphoma (CTCL) is a heterologous non‐Hodgkin’s lymphoma. Mycosis fungoides (MF) and Sezary syndrome are representative types of CTCL, but MF is the predominant form.^1^, ^2^, ^3^ MF mainly develops in middle‐aged and elderly people with a male : female ratio of 9:5.^4^, ^5^ CTCL presents as a skin disease characterized by patches and plaques, and tumors with or without peripheral adenopathy, and exhibits definite clinical stages. After the patch and plaque stages continue for 10 years or more, MF progresses to the tumor stage with the development of multiple tumors. Patients whose disease state has progressed to the tumor stage develop organ infiltration and infections, and have an extremely poor prognosis. During the course of the disease, the patient’s quality of life markedly decreases due to frequent occurrence or recurrence of the tumor mass, which affect the patient’s social life. Therefore, systematic treatment is important for preventing disease deterioration and improving the patient’s quality of life.

An annual survey of 1000 patients was started by the Minister’s Secretariat of Statistical Information Department in the Ministry of Health, Labor and Welfare in Japan, in 2011. In their 2014 and 2016 reports, only 145 and 161 people, respectively, were newly diagnosed with MF. Further, a survey on the number of patients with cutaneous lymphoma in Japan conducted by the Prognosis and Statistical Investigation Committee of the Japanese Skin Cancer Society registered a total of 1733 patients with primary cutaneous lymphoma from 2007 to 2011. Among these 1733 patients, 1485 had CTCL and 750 (43.3%) had MF.^6^ There are currently no treatments with high evidence levels, however, due in large part to the extremely small number of patients with CTCL, the difficulties in judging the success of therapeutic interventions due to gradual deterioration, and ethical issues involved in performing randomized controlled trials based on placebo. Nevertheless, due to the demands of physicians in clinical practice, the international standardization of diagnostic criteria and staging for CTCL has progressed, and the National Comprehensive Cancer Network and European Organization for Research and Treatment of Cancer have presented treatment guidelines.^7^, ^8^ In addition, a Clinical Practice Guideline describing a therapeutic policy by disease stage was issued in Japan.^9^ The report by Ibbotson^10^ in 2018 provides an invaluable perspective on the use of psoralen and ultraviolet (UV)‐A therapy (PUVA) and narrowband UV‐B for a range of skin diseases, including CTCL. In addition, a preliminary retrospective analysis performed by Shintani *et al.* (2020, pers. comm.) demonstrated the utility of PUVA in a study of 62 patients with CTCL treated with PUVA therapy, although treatment resistance in some patients led to a poor prognosis.

Vorinostat, a histone deacetylase inhibitor, and bexarotene (Targretin^TM^, 75 mg capsule, Minophagen Pharmaceutical Co., Ltd., Kanagawa, Japan) were approved as oral therapeutic agents for CTCL in 2011 and 2016 in Japan, respectively. In two studies evaluating early‐stage CTCL patients (L1069‐23 study) and advanced‐stage CTCL patients (L1069‐24 study), bexarotene at an initial dose of 300 mg/m^2^ provided an objective response (complete response [CR] + partial response [PR]) rate of 54.0% (15/28 patients) and 48.2% (25/56 patients), respectively, on the basis of the Physician Global Assessment of Clinical Condition (PGA).^11^, ^12^ A clinical phase I/II study (B‐1101 study) in 13 patients with CTCL in Japan reported a response rate (CR + PR) of 61.5% on the basis of the modified Severity‐Weighted Assessment Tool (mSWAT) at the time of completion of dosing (termination/discontinuation) after p.o. administration of bexarotene at an initial dose of 300 mg/m^2^.^13^ The objective response rate was 53.8% in the extension study (B‐1201 study) of the B‐1101 study.^14^ A clinical phase III study (NCT00056056 published in 2012) of the total response rate (CR + PR) to combination treatment with PUVA therapy and study drug administration compared with PUVA therapy alone in 93 patients with early CTCL confirmed no difference in the response rates between combination therapy and PUVA monotherapy, but the combination therapy tended to decrease the UV irradiation dose required compared with PUVA monotherapy.^15^ Reduction of the UV irradiation dose was also reported in one patient administrated oral etretinate combined with phototherapy.^16^ On this basis, we considered that combination therapy reduces resistance to phototherapy due to a decrease in the UV irradiation dose and suppresses phototherapy‐induced adverse drug reactions. The above‐described reports are based mainly on data of patients in Europe. Therefore, because of racial differences in photosensitivity due to variations in skin color, it is essential to conduct a clinical study of the combination therapy in Japanese patients. The present study investigated the efficacy and safety of combination therapy with phototherapy and bexarotene in Japanese CTCL patients.

## Methods

### Study design

This was a single‐arm, confirmatory, open‐labeled clinical study (UMIN000024532) of Japanese patients diagnosed with CTCL conducted at two sites, Nagoya City University and Osaka City University. This study was performed in accordance with protocols approved by the ethics review boards of Nagoya City University and Osaka City University, the Declaration of Helsinki and the Ethical Guidelines for Clinical Research.

### Subject patients

The major selection criteria were as follows, regardless of sex, hospitalization or outpatient status: Japanese patients at least 20 years of age who were diagnosed with CTCL, and provided written informed consent following an explanation of the study. The following exclusion criteria were applied: patients with contraindications to the investigational drug including severe hepatic impairment; medical history of hypersensitivity to the ingredients of the investigational drug; current treatment with a vitamin A preparation or vitamin A hypersensitivity; pregnant, lactating or wanting to become pregnant during the examination period; and patients judged to be ineligible for the study by a principal investigator or co‐investigator. The following items were examined/recorded on the treatment starting date: sex, birth date, definitive diagnosis date of CTCL, disease type and stage at the time of definitive diagnosis, major medical history, concomitant disease, height, bodyweight, body mass index, body surface, results of slit‐lamp microscope examination, chest X rays, and abdominal and pelvic computed tomography or positron emission tomography scan.

### Investigational therapy according to protocol

Bexarotene was administrated p.o. at 300 mg/m^2^ (body surface) once daily after meals. One of two types of UV irradiation (bath‐PUVA or narrowband UV‐B) was performed within 4 h after p.o. administration of the bexarotene. For the bath‐PUVA, irradiation was started with 0.5 J/cm^2^ UV‐A , and carried out five times a week for 4 weeks after beginning the bexarotene administration. The irradiation dose was increased by 0.5 J/cm^2^ at each irradiation session (maximum, 4.0 J/cm^2^). From 4 weeks after the initiation of bexarotene administration, if the principal investigator or co‐investigator judged that there was no problem related to subject safety and the disease conditions improved, the irradiation dose or number of irradiations was changed. For the narrowband UV‐B, treatment was started from 50% to 70% of the minimum erythema dose or 0.5 J/cm^2^ to 0.7 J/cm^2^ within 4 h after p.o. administration of the bexarotene. Irradiation was performed five times a week for 2 weeks after beginning bexarotene administration, and the irradiation dose was increased by 20% at each irradiation session (maximum, 2.0 J/cm^2^). From 2 weeks after the initiation of bexarotene administration, when the principal investigator or co‐investigator judged that there was no problem related to subject safety and the disease conditions improved, the irradiation dose or the number of irradiations was changed.

### Assessment of efficacy and safety

The following items were measured or surveyed at the first day of therapy in weeks 1, 2, 3, 4, 8, 12 and 24, or discontinuation day: mSWAT, PGA, safety, hematology tests (white blood cells [including differentiation], red blood cells, hemoglobin, hematocrit, platelets), blood biochemical examination (alanine aminotransferase, aspartate aminotransferase, total bilirubin, alkaline phosphatase, creatinine phosphokinase, Na, K, Cl, Ca, P, Mg, lactate dehydrogenase [LDH], triglycerides, total cholesterol, high‐density lipoprotein, low‐density lipoprotein, total protein, albumin, uric acid, S‐amylase, thyroid‐stimulating hormone, triiodothyronine, thyroxine), coagulation system (prothrombin, activated partial thromboplastin time), C‐reactive protein, fasting blood sugar, hemoglobin A1c, soluble interleukin‐2 receptor (sIL‐2R; excluding weeks 1 and 3), serum thymus and activation‐regulated chemokine (TARC; excluding weeks 1, 2 and 3, including weeks 4 and 8 when possible), body temperature, blood pressure, pulse rate, concomitant medication and therapy, subjective symptoms, objective findings and drug compliance status. The response was defined as follows: CR, 100% clearance of skin lesions; PR, 50–99% clearance of skin disease from baseline without new tumors (T3) in patients with T1‐, T2‐ or T4‐only skin disease; stable disease, less than 25% increase to less than 50% clearance in skin disease from baseline without new tumors (T3) in patients with T1‐, T2‐ or T4‐only skin disease; progressive disease, 25% increase in skin disease from baseline or new tumors (T3) in patients with T1‐, T2‐ or T4‐only skin disease; and loss of response, a skin score increase greater than the sum of the nadir plus 50% of the baseline score in patients with complete or partial response.^17^

### Statistical analysis

Statistical analysis was performed using JMP version 13.2 (SAS Institute, Cary, NC, USA) and Excel (Microsoft, Redmond, WA, USA). For continuous variables, we calculated summary statistics (number of patients, mean, standard deviation, minimum, median and maximum). For discrete variables, we calculated the number of subject cases and their ratios. Furthermore, efficacy was assessed by mSWAT and PGA at the scheduled time points up to week 24 after the start of treatment. Safety assessment including clinical examination was also conducted up to week 24.

## Results

### Patient profile

**Figure 1:**
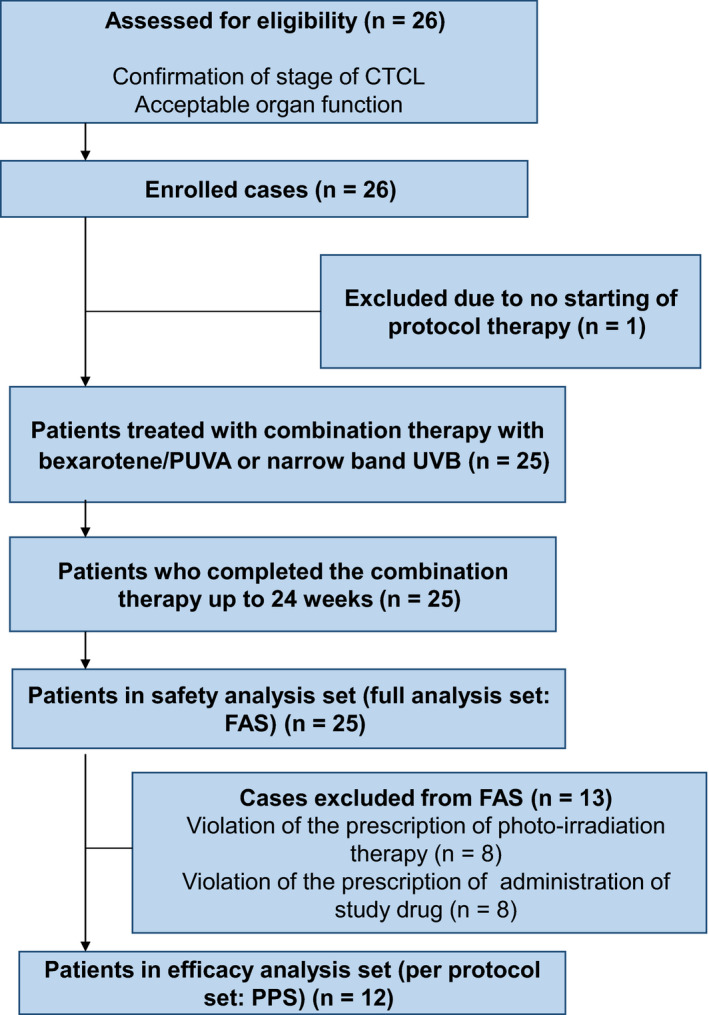
Study design and patient flow. CTCL, cutaneous T‐cell lymphoma; FAS, full analysis set; PPS, per protocol set; PUVA, psoralen and ultraviolet A therapy; UVB, ultraviolet B.

Research design and patient flow are shown in Figure 1. A total of 26 Japanese subjects were registered at two research sites, but one patient was excluded from the study because the treatment protocol was not started. The remaining 25 patients completed the study without discontinuation/suspension of treatment and constituted the full analysis set (FAS). As 13 of the 25 patients in the FAS violated the UV therapy or drug administration protocol or both, 12 patients constituted the per protocol set (PPS). Table 1 shows the patient background at the time of diagnosis confirmation in the FAS (*n* = 25) and PPS (*n* = 12). The mean age ± standard deviation of patients in the FAS was 69.1 ± 13.2 years, with 11 (44.0%) men and 14 (56.0%) women. For the PPS, the mean age was 67.6 ± 12.3 years, with six men and six women (50% each). The mean duration of CTCL ± standard deviation was 3.5 ± 4.7 years for FAS and 3.1 ± 3.7 years for PPS.

**Table 1:** Baseline characteristics of CTCL patients

Category	Summary statistics	Safety/FAS (%)	PPS (%)
		No. of subjects (%), total *n* = 25	No. of subjects (%), total *n* = 12
Sex	Male/female	11 (44.0)/14 (56.0)	6 (50.0)/6 (50.0)
Age category (years)	Mean value ± SD	69.1 ± 13.2	67.6 ± 12.3
	Median	75.0	68.5
	Range	41–89	44–84
	<50	3 (12.0)	1 (8.3)
	≥50 to <60	4 (16.0)	3 (25.0)
	≥60 to <70	2 (8.0)	2 (16.7)
	≥70 to <80	13 (52.0)	5 (41.7)
	≥80	3 (12.0)	1 (8.3)
Duration of CTCL (years)	Mean value ± SD	3.5 ± 4.7	3.1 ± 3.7
	Median	1.8	1.7
	Range	0.1–20.0	0.1–10.7
Type of CTCL	Mycosis fungoides	25 (100.0)	12 (100.0)
	Sezary syndrome	0 (0.0)	0 (0.0)
Disease phase at definite diagnosis	IA	2 (8.0)	2 (16.7)
	IB	10 (40.0)	3 (25.0)
	IIA	1 (4.0)	1 (8.3)
	IIB	2 (8.0)	1 (8.3)
	IIIA	8 (32.0)	4 (33.3)
	IIIB	0 (0.0)	0 (0.0)
	IVA	0 (0.0)	0 (0.0)
	IVA2	1 (4.0)	1 (8.3)
	IVB	0 (0.0)	0 (0.0)
	Unevaluable	1 (4.0)	0 (0.0)
	IA to IB	12 (48.0)	5 (41.7)
	From IIA	12 (48.0)	7 (58.3)
	Unevaluable	1 (4.0)	0 (0.0)
	To IIA	13 (52.0)	6 (50.0)
	From IIB	11 (44.0)	6 (50.0)
	Unevaluable	1 (4.0)	0 (0.0)
Presence or absence of medical history/complication	No	0 (0.0)	0 (0.0)
	Yes	25 (100.0)	12 (100.0)
Body surface area (m^2^)	Mean value ± SD	1.58 ± 0.18	1.63 ± 0.18
	Median	1.61	1.63
	Range	1.30–1.87	1.30–1.86
	<0.88	0 (0.0)	0 (0.0)
	0.88–1.12	0 (0.0)	0 (0.0)
	1.13–1.37	6 (24.0)	2 (16.7)
	1.38–1.62	8 (32.0)	4 (33.3)
	1.63–1.87	11 (44.0)	6 (50.0)
	>1.88	0 (0.0)	0 (0.0)
Slit‐lamp microscopy	Normal	7 (28.0)	5 (41.7)
	Abnormal	17 (68.0)	7 (58.3)
	Cataract	15 (60.0)	6 (50.0)
	Others	3 (12.0)	1 (8.3)
	Unclear (no examination)	1 (4.0)	0 (0.0)

CTCL, cutaneous T‐cell lymphoma; FAS, full analysis set; PPS, per protocol set; SD, standard deviation.

For the disease types (ratio), all of the patients had MF, accounting for 100.0% of the FAS (25 patients) and 100.0% of the PPS (12 patients), and no patients had Sezary syndrome. In the FAS, the disease stage at the definitive diagnosis period was IA to IB in 12 patients (48.0%), IIA or higher in 12 patients (48.0%) and unable to be assessed in one patient (4.0%). In the PPS, five patients (41.7%) were stage IA to IB and seven patients (58.3%) were stage IIA or higher. The mean body surface (± standard deviation) value in the FAS was 1.58 ± 0.18 m^2^ and that in the PPS was 1.63 ± 0.18 m^2^.

All patients had a previous medical history and experienced complications. Major complications were common in the FAS and PPS: cataract (FAS, 14 patients [56.0%]; PPS, five [41.7%]), hypertension (FAS, 13 patients [52.0%]; PPS, six [50.0%]), osteoporosis, insomnia, constipation and hypercholesterolemia (FAS, 7–8 patients [28.0–32.0%]; PPS, 3–4 patients [25.0–33.3%]). Cataracts were most frequently observed in the medical history and were reported in four (16.0%) patients in the FAS and three patients (25.0%) in the PPS. Next, in the FAS, three patients (12.0%) suffered from cerebral infarction, while in the PPS, appendicitis, inguinal hernia and lumbar spinal canal stenosis were observed in two patients (16.7%).

### Efficacy

**Figure 2:**
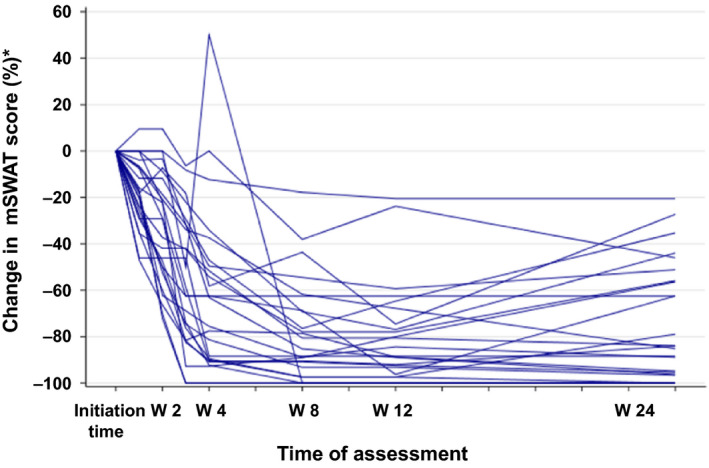
Percentage change of overall cutaneous lesions based on the modified Severity‐Weighted Assessment Tool (mSWAT) score. Changes in the improvement rate are indicated by the mSWAT score for each patient.

**Figure 3:**
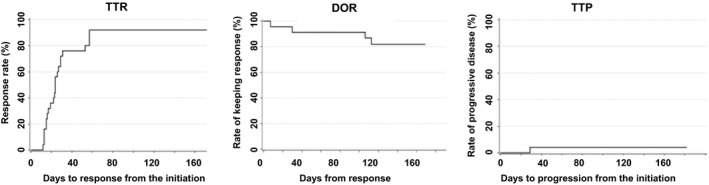
Time to response (TTR), duration of response (DOR) and time to progression (TTP) of full analysis set (*n* = 25).

**Figure 4:**
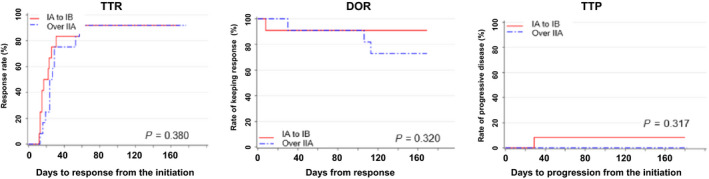
Time to response (TTR), duration of response (DOR) and time to progression (TTP) of patients stratified to stages IA to IB and IIA and over IIA. *n* = 24 (*n* = 12 in each stage category); log–rank test, no significant difference between the stages from IA to IIA and over IIA.

**Figure 5:**
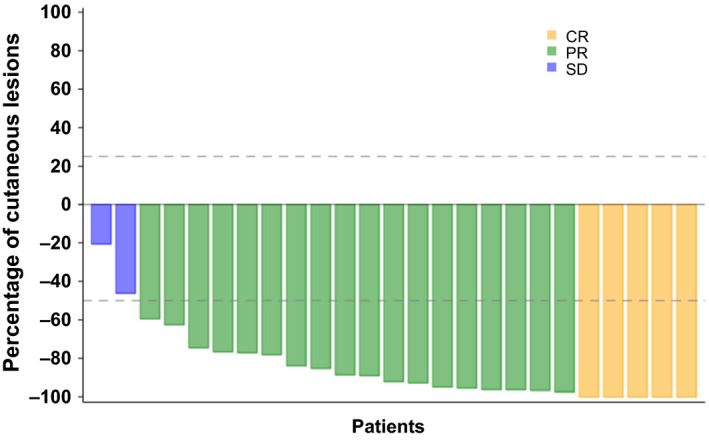
Best overall responses in overall cutaneous lesions based on the modified Severity‐Weighted Assessment Tool (mSWAT) scores. Best overall responses in overall cutaneous lesions were assessed by the mSWAT. CR, complete response; PR, partial response; SD, stable disease.

Table 2 shows the assessment of general cutaneous lesions at week 24 by the mSWAT. The assessment confirmed a high overall response (CR + PR) rate of 80.0% (20/25 patients) in the FAS and 75.0% (9/12 patients) in the PPS. Although none of the patients exhibited progressive disease, five (20%) patients had stable disease in the FAS and three (25%) had stable disease in the PPS. The results determined by the PGA at week 24 are shown in Table 3. The overall response rate, including 16.0% of clinical complete response (CCR), achieved 84.0% in the FAS and 83.3% (CCR, 16.7%) in the PPS. In assessments using both the mSWAT and PGA, no patient had a disease state that was aggravated. The change in the assessment according to the mSWAT of individual patients up to week 24 is illustrated in Figure 2. In most patients, combination therapy rapidly improved the cutaneous lesions up to week 4, and the ratio of the cutaneous lesions evaluated according to the mSWAT reached 37.9 ± 35.5% in the FAS. Subsequent improvements were slow, with a mean of 20–26%. Evaluation by the PGA showed the same tendency. Time to response (TTR), duration of response (DOR) and time to progression (TTP) in the FAS evaluated by the mSWAT were 20.7 ± 9.62, 117.0 ± 43.0 and 163.6 ± 28.8 days, respectively (Table 4, Fig. 3). TTR, DOR and TTP were evaluated after stratifying the patients to disease stages IA to IB and IIA or higher (Fig. 4). The log–rank test confirmed that there were no statistically significant differences in TTR, DOR and TTP (*P* = 0.380, 0.320 and 0.317, respectively) between the two categories. The same result was obtained when stratifying the patients as IA to IIA and IIB or higher. Best overall responses in cutaneous lesions of the patients in the FAS were evaluated by the mSWAT, and the percent change by individual is shown in Figure 5 using waterfall plots. A CR was achieved by five (20.0%) patients, a PR by 18 (72.0%) and the remaining two patients had stable disease (the ratio of not improving cutaneous lesions was 80% and 50%). The results evaluated by the PGA and mSWAT were similar.

**Table 2:** Assessment of global cutaneous legion at week 24 after treatment initiation by mSWAT

Results of efficacy	FAS (*n* = 25)		PPS (*n* = 12)	
	No. of patients	No. of responders and non‐responders	No. of patients	No. of responders and non‐responders
Responders				
CR	5 (20.0%)	CR + PR 20 (80.0%)^†^	3 (25.0%)	CR + PR 9 (75.0%)^‡^
PR	15 (60.0%)		6 (50.0%)	
Non‐responders				
SD	5 (20.0%)	SD + PD 5 (20.0%)	3 (25.0%)	SD + PD 3 (25.0%)
PD	0 (0.0%)		0 (0.0%)	

CR, complete response; FAS, full analysis set; mSWAT, modified Severity‐Weighted Assessment Tool; PD, progressive disease; PPS, per protocol set; PR, partial response; SD, stable disease.

^†^ 95% confidence interval, 59.3–93.2%.

^‡^ 95% confidence interval, 42.8–94.5%.

**Table 3:** Assessment of global cutaneous legion at week 24 after treatment initiation by PGA

Judgement/result of efficacy	FAS (*n* = 25)		PPS (*n* = 12)	
	No. of patients	Results of efficacy/no. of patients	No. of patients	Results of efficacy/no. of patients
Responders				
Completely disappeared CCR	4 (16.0%)	CCR + PR 21 (84.0%)^†^	2 (16.7%)	CCR + PR 10 (83.3%)^‡^
Almost disappeared PR	5 (20.0%)		3 (25.0%)	
Significantly improved PR	9 (36.0%)		3 (25.0%)	
Moderately improved PR	3 (12.0%)		2 (16.7%)	
Non‐responders				
Mildly improved SD	4 (16.0%)	SD + PD 4 (16.0%)	2 (16.7%)	SD + PD 2 (16.7%)
Not changed SD	0 (0.0%)		0 (0.0%)	

CR, complete response; FAS, full analysis set; PD, progressive disease; PGA, Physician Global Assessment; PPS, per protocol set; PR, partial response; SD, stable disease.

^†^ 95% confidence interval, 63.9–95.5%.

^‡^ 95% confidence interval, 51.6–97.9%.

**Table 4:** Time to response, time to progression and duration of response by mSWAT

	TTR	DOR	TTP
No. of cases	20	20	25
Mean ± SD (days)	20.7 ± 9.62	117.0 ± 43.0	163.6 ± 28.8
Q1/median/Q3 (day)	13.0/20.0/27.0	68.0/140.0/154.0	164.0/168.0/174.0
Range (days)	13.0–55.0	56.0–154.0	29.0–182.0

DOR, duration of response; mSWAT, modified Severity‐Weighted Assessment Tool; SD, standard deviation; TTP, time to progression of disease; TTR, time to response.

### Biologic markers

**Figure 6:**
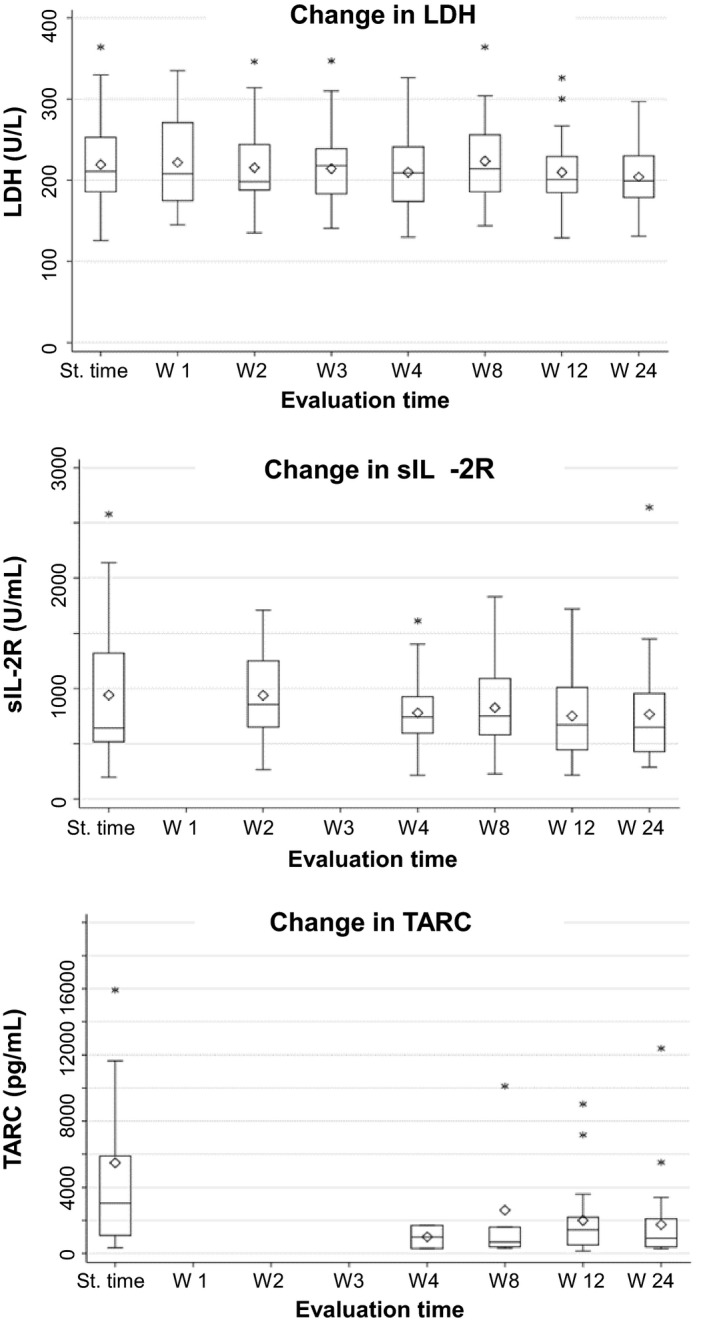
Box‐and‐whisker plots of lactate dehydrogenase (LDH), soluble interleukin‐2 receptor (sIL‐2R) and serum thymus and activation‐regulated chemokine (TARC, T‐helper 2 chemokine). St. time, starting time.

Thymus and activation‐regulated chemokine is a biomarker for MF and Sezary syndrome.^18^ As among the clinical test items, LDH, sIL‐2R and TARC are positively correlated with malignant lymphoma, we also evaluated the changes in these items in relation to the present treatment. The changes in LDH, sIL‐2R and TARC are shown as boxplots in Figure 6. The TARC values were significantly lower at weeks 12 and 24, compared with the starting point (*P* = 0.004 and 0.001, respectively; Wilcoxon signed‐rank test), but significant changes were not observed for LDH and sIL‐2R. For these clinical test items, we evaluated the effect of disease stage by stratifying the patients into stages IA to IB and stages IIA or higher; the results indicated no difference between the stages for LDH and sIL‐2R, but TARC levels at weeks 12 and 24 of the patients at stage IIA or higher were significantly lower than that at the starting time point (*P* = 0.027 and 0.016, respectively; Wilcoxon signed‐rank sum test; data not shown).

### Safety

In this clinical study, all patients experienced adverse events with a total of 151 adverse events (adverse drug reactions) reported for all of the patients. Adverse events that developed in three (12.0%) or more patients are summarized in Table 5. Adverse events with a high incidence were as follows: hypertriglyceridemia (23 events in 21 patients, 84.0%), hypothyroidism (22 events in 21 patients, 84.0%), neutropenia (16 events in 15 patients, 60.0%), hypercholesterolemia (15 events in 14 patients, 56.0%), anemia (eight events in eight patients, 32.0%), and nasopharyngitis and burns (six events in five patients, 20.0%). Among the adverse events, severe events of at least grade 3 included sepsis (*n* = 1), anemia (*n* = 1), congestive cardiac insufficiency (*n* = 1), pyrexia (*n* = 1) and fracture around an artificial joint (*n* = 2). Hyperlipidemia and neutropenia of grade 3 or higher, which often lead to a dose reduction or interruption of bexarotene, did not develop in this study, probably due to the addition of PUVA. The severity of other adverse drug reactions was mild to moderate. In a total of 19 cases, the bexarotene dose had to be reduced or discontinued due to the onset of an adverse event.

**Table 5:** Adverse events observed in three or more cases during the course of combined therapy with bexarotene and ultraviolet irradiation

Adverse events	No. of adverse events	No. of patients (%)
Whole	151	25 (100.0)
Metabolism and nutrition disorders	43	22 (88.0)
Hypertriglyceridemia	23	21 (84.0)
Hypercholesterolemia	15	14 (56.0)
Endocrine disorders	22	21 (84.0)
Hypothyroidism	22	21 (84.0)
Blood and lymphatic system disorders	34	19 (76.0)
Neutropenia	16	15 (60.0)
Anemia	8	8 (32.0)
Thrombocytosis	4	4 (16.0)
Neutrophilia	3	3 (12.0)
Leukopenia	3	3 (12.0)
Infections and infestations	15	12 (48.0)
Epipharyngitis	6	5 (20.0)
Injury, poisoning and procedural complications	7	5 (20.0)
Burn injury	6	5 (20.0)

## Discussion

The treatment strategy for malignant lymphoma is determined by the pathological diagnosis, disease stage classification and prognosis assessment, similar to other malignant tumors. The available therapeutic methods include systemic and local therapies, such as topical application, phototherapy and radiation therapy. Single‐agent therapy, multidrug combination therapy and combined therapy are performed as systemic therapies. The bexarotene used in this study is an anticancer agent classified as a retinoid that can be applied to all treatment‐resistant CTCL, and has a high therapeutic effect, even when used as a single agent. Furthermore, Duvic *et al.* 12 reported that the response rate of MF patients in stages IA to IIA administrated 300 mg/m^2^ bexarotene alone was 54% with a CR rate of 7%. In another study of patients with refractory advanced phase MF/Sezary syndrome (IIB to IVB), Duvic *et al.* 11 reported a response rate to bexarotene of 45%, a CR rate of 2% and a median DOR of 299 days.

On the other hand, the UV phototherapy (e.g. PUVA, narrowband UV‐B and excimer light) used in this combination therapy is a local therapy together with steroid application directly to the skin. This therapy is widely used to control disease progression in patients with early MF (stage IA, IB and IIA). Ahmad *et al.* 19 conducted a retrospective study on UV radiation therapy for patients with early‐stage MF, and reported that narrowband UV‐B therapy results in a CR in 50% (6/12 patients) and a PR in 33% (4/12 patients), while PUVA yields a CR in 64% (18/28 patients) and a PR in 21% (6/28 patients).^19^ In another study by Whittaker *et al.*,^20^ UV irradiation treatment in early‐stage disease produced good results with a stage progression rate in the IA and IB phases of 9–20%.

We evaluated combination therapy with bexarotene and PUVA for the first time in Japanese patients. Although the disease phase was different between our study and the studies described above, the response rate as determined by the mSWAT reached 80% (20/25 patients) in the FAS and 75% (9/12 cases) in the PPS; even when limiting the disease stage to IIA or higher, the response rate was 75% (9/12 patients) in the PPS. Thus, this combination therapy showed a higher response rate than bexarotene monotherapy. The high response rate in this study is probably attributable to: (i) the inclusion of many early‐stage MF patients; and (ii) topical application of various steroids. Furthermore, although clear evidence was not obtained, we hypothesized that the combined use of UV irradiation would improve the response rate. The study conditions between the above‐described monotherapy and our combination therapy are very different. Therefore, it is impossible to statistically compare the studies. Our findings, however, suggest that combination therapy is a valid option for MF therapy. The study performed by Whittaker *et al.*,^20^ comparing combination therapy with bexarotene plus PUVA and PUVA monotherapy on European MF patients with stages IB to IIA supports the validity of combination therapy for MF.^15^ Although they reported no statistically significant difference between the two groups partly due to the small number of patients in the study, the best overall response rate was 71% for the PUVA monotherapy and 77% for the combination therapy. The total number of patients in stages IA and IB in the present study was 12 in the FAS, and the CR and PR in the best overall evaluation were 42% and 50%, respectively. One patient showed stable disease, and the stage progression rate was 8.3%. This result is comparable with the study results of both Ahmad *et al.* 19 and Whittaker *et al.* 15 We could not conclude, however, that the effectiveness of the present combination therapy was superior to the UV irradiation monotherapy.

A comparison between the combination therapy with bexarotene plus PUVA and PUVA monotherapy performed by Whittaker *et al.* 15 indicated that the combination therapy tends to decrease the UV irradiation dose or irradiation frequency or both. Although the efficacy of the combination therapy and monotherapy was not compared in the present study, the irradiation dose and irradiation frequency were not increased compared with other studies on UV irradiation therapy. In this clinical study, 20 of 25 patients (80%) had a CR or PR, but the integrated irradiation dose received by 25 patients was 104.5 ± 63.2 J/cm^2^ in whole‐body PUVA, 66.9 ± 41.8 J/cm^2^ for the limb type PUVA and 12.8 ± 2.1 J/cm^2^ for the narrowband UV‐B. In the Whittaker *et al.* 15 comparison of combination therapy with bexarotene plus PUVA and PUVA monotherapy, the mean integrated irradiation dose was 107 J/cm^2^ for PUVA monotherapy and 101.7 J/cm^2^ for combination therapy, which is almost equivalent to those in the present study. That is, the reactivity of Japanese patients to the combination therapy of bexarotene and PUVA is unlikely to differ significantly from that of patients living in Europe and the USA.

All 25 patients in our study experienced adverse events and adverse drug reactions during the course of treatment (Table 5). A total of 151 adverse events was observed; hypertriglyceridemia (84.0%) and hypothyroidism (84.0%) occurred most often, followed by neutropenia (60.0%) and hypercholesterolemia (56.0%). Hypertriglyceridemia (84.0%) and hypothyroidism (84.0%) as adverse drug reactions had the highest incidence. Neutropenia (60.0%) and hypercholesterolemia (56.0%) had the next highest incidence, followed by anemia (32.0%). These have been reported as adverse drug reactions of bexarotene both in Japan and elsewhere, and none were specific to this study. Bexarotene exhibits phototoxicity in *in vitro* tests (photo‐hemolytic test and histidine photo‐oxidation reaction).^14^ Non‐serious photosensitivity was reported in combination therapy with UV irradiation (UV‐B therapy) in 6.3% of patients (1/16 patients) in a phase I/II study (B‐1101 study) in Japan,^13^, ^14^ and 1.7% (1/59 patients) in a phase IV study (E7273‐G000‐401 study) (2019, pers. comm.) and 1.7% (1/58 patients) in a phase II/III study (L1069‐23 study) performed in the USA.^11^ In the present study, there were no reports of photosensitivity, neither was there a case of interruption/discontinuation of the study due to adverse events and adverse drug reactions.

These results confirm that combination therapy with bexarotene and PUVA is safe and markedly effective in Japanese CTCL patients.

## Conflict of Interest

This research was funded by Minophagen Pharmaceutical, Tokyo, Japan, upon implementation.
